# A Comparison of Growth and Development of Three Major Agricultural Insect Pests Infected with Heliothis virescens ascovirus 3h (HvAV-3h)

**DOI:** 10.1371/journal.pone.0085704

**Published:** 2013-12-30

**Authors:** Shun-Ji Li, Xing Wang, Zhong-Shi Zhou, Jie Zhu, Jue Hu, Yi-Pei Zhao, Gui-Wei Zhou, Guo-Hua Huang

**Affiliations:** 1 Institute of Virology, Hunan Agricultural University, Changsha, Hunan, China; 2 Department of Plant Protection, Oriental Science & Technology College of Hunan Agricultural University, Changsha, Hunan, China; 3 Institute of Plant Protection, Chinese Academy of Agricultural Sciences, Beijing, Beijing, China; Ecole des Mines d'Alès, France

## Abstract

Ascoviruses are double-stranded DNA viruses that are pathogenic to lepidopteran hosts, particularly noctuid larvae. Infection of a larva is characterized by retarded growth, reduced feeding and yellowish body color. In this paper, we reported the growth and development of three major agricultural noctuid insect pests, *Helicoverpa armigera* (Hübner), *Spodoptera exigua* (Hübner) and *Spodoptera litura* (Fabricius), infected with Heliothis virescens ascovirus 3h (HvAV-3h). Using 10-fold serial dilutions (0 to 7) of HvAV-3h-containing hemolymph to infect *S. litura* larvae, we found no significant difference in larval mortalities from 0 to 10^3^-fold dilutions; however, significant differences were observed at 10^4^-fold dilution and above. Using a 10-fold dilution of HvAV-3h-containing hemolymph to infect *H. armigera*, *S.* exigua and *S. litura* larvae, we found that the growth and development were significantly affected. All infected larvae could not pupate; the survival times of treated *H. armigera*, *S.* litura and *S. exigua* larvae were significantly longer than untreated control larvae. Body weight showed significant difference between treated and untreated control group from day 1 after inoculation in *H. armigera* and *S. exigua*, but day 2 in *S. litura*. Additionally, food intake also showed significant difference between treated and untreated control group from day 2 after inoculation in *H. armigera* and *S. litura*, but day 3 in *S. exigua*.

## Introduction

Ascoviruses (AVs), belonging to the family of *Ascoviridae*, are recently discovered insect-specific viruses with a circular double-stranded DNA genome of 100-199 kbp [1-3]. These viruses were first isolated from lepidopteran larvae in the late 1970s [4] and named in the early 1980s [5-7]. Ascovirus-infected lepidopteran larvae are characterized by the stunted growth with flavescent in body color, and it is difficult to recognize and discover the virus in field condition [6,8-11].

Once in a laboratory condition, larvae infected by ascovirus are easier to recognize through checking the hemolymph that is milky-white in color, which is opposite to the transparent hemolymph in healthy larvae [7,8]. This infection symptom in larvae is distinguishable from other viruses, such as baculovirus. 

The milky-white hemolymph is attributed to the formation of virion-containing vesicles [10,12]. These vesicles contain large enveloped virions with 130×400 nm in size. The virion shape is similar to baculoviruses but more variable, from bacilliform to ovoid or allantoid [7,13]. Indeed, the virion shape and the hemolymph vesicles aid in ascovirus identification [14].

Since their early isolation in the USA, ascoviruses have been reported from Indonesia, Australia, France and China [4,6,10,15-17]. These ascoviruses have been assigned to four species according to the International Committee on Taxonomy of Viruses (ICTV): *Spodoptera frugiperda ascovirus 1a* (SfAV-1a), *Trichoplusia ni ascovirus 2a* (TnAV-2a), *Heliothis virescens ascovirus 3a* (HvAV-3a), and *Diadromus puchellus ascovirus 4a* (DpAV-4a) [18]. The most recently discovered and reported ascovirus is Heliothis virescens ascovirus 3h (HvAV-3h), which was isolated from a *Spodoptera exigua* larva in China [10].

The effects of AV infection on larval growth and development were studied using *Trichoplusia ni*, *Spodoptera frugiperda*, *Helicoverpa zea*, and *Heliothis virescens* larvae in the early 1990s [19]. Although these studies reported that AV-infected larvae were observed stunted growth and gained little weight before dying of the disease, it remains unclear whether this is a general phenomenon [5,8]. Therefore, the physiological changes of the larvae were evaluated based on the recently discovered HvAV-3h isolate in this work.

In order to understand how the body weight changes with food intake, and how the HvAV-3h affects larval growth and development, we selected *Helicoverpa armigera*, *S.* exigua and *Spodoptera litura* to test the body weight, food intake of the larvae. Our results provide information for further investigation of how HvAV-3h infection regulates the growth and development of different insects. 

## Materials and Methods

### Insects and viruses

The S. *exigua* eggs were kindly provided by Dr. Yue-Qin Song of Henan University of Science and Technology, the *H. armigera* larvae were donated by Dr. Zhu-Dong Liu of Chinese Academy of Sciences, and the S. *litura* larvae were collected from a vegetable field (an experimental field of our laboratory) near Hunan Agricultural University, Changsha, China. The S. *exigua* larvae were reared on artificial diets following Song’s method [20], and the *H. armigera* and *S. litura* larvae were reared on pinto bean-based diets [21]. The larvae of the three species were maintained in an incubator with a controlled temperature at 27±1°C, relative humidity at 70%, and a light:dark photoperiod at 14:10-h [20]. The insect adults were supplied with 10% honey solution.

The isolate HvAV-3h has been previously described in our laboratory [10]. A laboratory stock of HvAV-3h was amplified through the inoculation of third-instar *S. litura* larvae with HvAV-3h-containing hemolymph by pinning a proleg [11,17]. At day 6 post-inoculation, HvAV-3h-containing hemolymph was harvested by cutting one proleg of the infected *S. litura* larvae [11,19]. The virus-containing hemolymph was used immediately or stored at -20°C. 

### Ascovirus titer determination

The concentration of HvAV-3h genomes in the hemolymph obtained from infected insects was estimated by adapting the end-point dilution method [22,23]. A stock of purified HvAV-3h DNA (20 ng/µl) [10] was 10-fold serially diluted with sterile water, a total of seven solutions were made (10^0^- to 10^6^- fold dilution). The HvAV-3h-containing hemolymph was also diluted similarly. To investigate the detective performance of both the purified DNA sample and the HvAV-3h-containing hemolymph, 1 µl each of DNA and HvAV-3h containing-hemolymph from each sample was added to PCR with a pair of ascovirus polymerase gene primers (polF: 5’-CCAGGATCACCAACACAC-3’, polR: 5’-GCTAGAGGATCGCTAACG-3’) using the conventional Taq enzyme based amplification reaction (94°C-1 min, (94°C-1 min, 55°C-1 min, 72°C-1 min) ×30 cycles, 72°C-10 min). After the reaction, the PCR products were analyzed by agarose gel electrophoresis. The PCR provided an all-or-none end-point at a very low DNA. Then the HvAV-3h genome copy was determined based on the comparison of HvAV-3h-containing hemolymph dilutions with HvAV-3h DNA dilutions that could not produce detectable PCR band in the agarose gel electrophoresis. 

Here, we considered that each copy of HvAV-3h DNA represents an infectious unit. The titer of HvAV-3h-containing hemolymph is a function of HvAV-3h genome copy number in hemolymph and the hemolymph volume, where the genome copy number is defined as a ratio of the total copy numbers over the genomic size (164 kbp) [10], based on the average molecular weight of one base-paired nucleotide (660 g/mol) and Avogadro’s constant (6.022×10^23^) [24].

### Dose-response assay

Serial dilutions (10-fold) of HvAV-3h-containing hemolymph were used to prepare eight inocula (10^0^- to 10^7^- fold dilution) with sterile water. Thirty third-instar (L3) *S. litura* larvae were used for each dilution, with thirty L3 larvae used as an untreated control (healthy hemolymph with corresponding dilutions). The whole experiment was done in triplicate. Inoculation was performed by pinning a proleg of each larva with a mini-pin tip contaminated with the respective HvAV-3h dilution, and the virus-killed larvae were collected each day to calculate the mortality rate of each dilution. 

### Survival time and molting period

A 10-fold dilution was chosen to assess the effects of HvAV-3h infection on *H. armigera*, *S.* exigua, and *S. litura* larvae. To each species, a total of 60 L3 larvae of each species were randomly chosen: 30 were inoculated with 10-fold dilutions of HvAV-3h, and the other 30 were inoculated with 10-fold dilutions of hemolymph from healthy larvae. Another two repeats were included to confirm the experiment, and a blank control without larvae was also included to calculate the evaporation rates. 

After inoculation, all the tested larvae were transferred to test tubes (2×10 cm) containing a 1-cm^3^-diet cake. In order to analyze the survival time and ecdysis between groups, the larvae were monitored every day until die (treated larvae) or adults’ emergence (untreated control larvae). Molting was recorded by checking exuviation and head capsule of the larva. Every day, the dead individuals were collected to calculate survival time and mortality rate.

### Larval feeding and growth

Following the above, each larva was weighted separately using an electronic balance, and the diet cakes were weighted after removing the feces with a camel brush. The diet cakes were discarded and replaced with a fresh one every 3 days. The blank control containing only diet cakes were weighted every day as well. The evaporation rate was then calculated and used in the data. So, daily data of body weight and food intake was recorded for each larva in the experiment.

### Statistical analyses

Larval mortality data were arcsine square-root-transformed to meet homoscedasticity assumptions [25,26], and the data of larval body weight and food intake were checked with the test of homogeneity. Then a one-way analysis of variance (ANOVA) was used to analyze the mortalities caused by different dilutions (with a post-hoc analysis) and the data of larval body weight and food intake between treated and untreated control groups [27]. Mortalities of the three species caused by HvAV-3h were checked by non-parametric test. Survival curves of *H. armigera*, *S.* exigua and *S. litura* larvae as well as median survival time (ST_50_, referring here as larval stage time) were obtained through Kaplan-Meier method (SPSS) [28], untreated control larvae data accounted from the day of inoculation to adults emergence. 

## Results

### HvAV-3h titration

**Figure 1:**
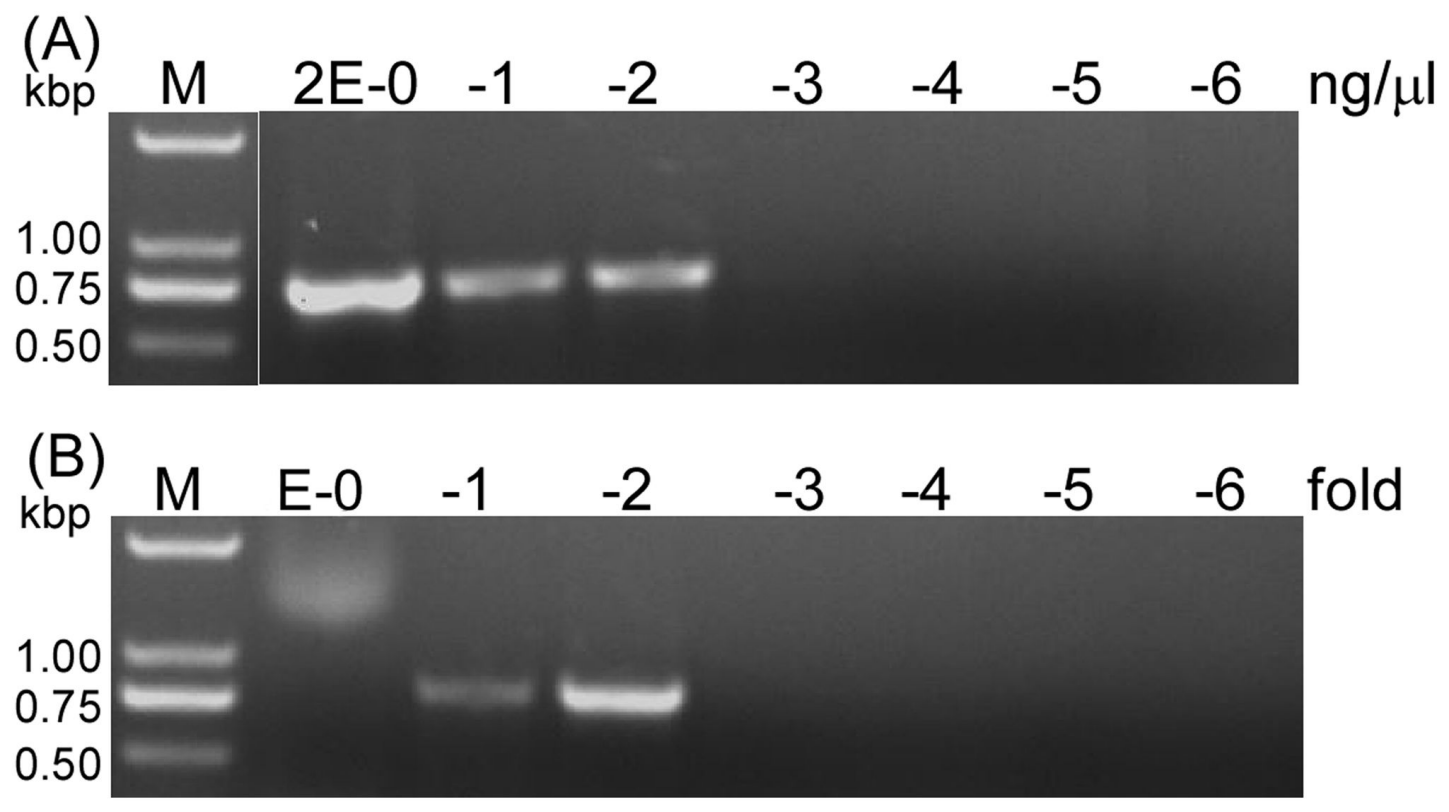
HvAV-3h end-point titer. Purified HvAV-3h DNA sample (A) and HvAV-3h-virion-containing hemolymph sample (B). Lanes with different numbers represent different dilution (from 10^0^- to 10^6^-fold).

Undiluted HvAV-3h-containing hemolymph did not result in PCR amplification, where 10^1^- and 10^2^-fold dilution showed discernible PCR bands (Figure 1B). There was no detectable band in the lanes of 10^3^-fold dilution and above. This result indicated that undiluted HvAV-3h-containing hemolymph (probably including some PCR inhibitors) could not be directly used as template in a PCR for the viral DNA amplification, and the 10^2^-fold diluted HvAV-3h-containing hemolymph (maybe the PCR inhibitors being diluted away) could be used directly as template. 

Using the purified HvAV-3h DNA dilutions as templates, the PCR had a detection limit at 0.2 ng (Figure 1A), whereas the HvAV-3h-hemolymph dilution had a detection limit at 10^2^-fold (Figure 1B). The result suggested that the viral DNA concentration in the 10^2^-fold purified HvAV-3h DNA sample was similar to the 10^2^-fold dilution of HvAV-3h-hemolymph. Based on this result, the titer of HvAV-3h-containing hemolymph was 1.1×10^11^ genome copies/ml according to the calculation showed above. The titer was considered be a relative concentration, and was used for the dose-response experiment.

### Dose-response assay

**Figure 2:**
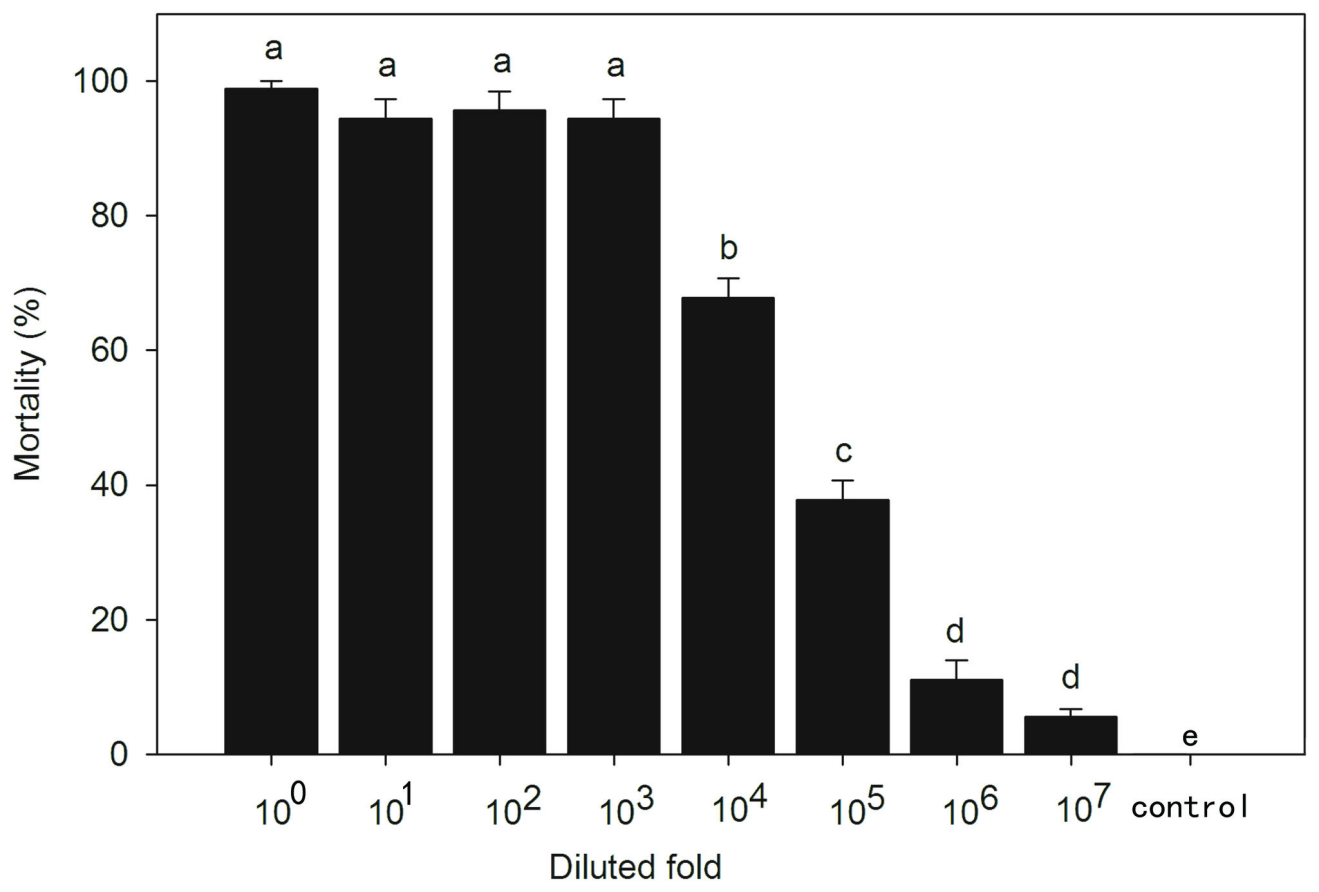
Mortalities of *Spodoptera litura* larvae caused by different diluted HvAV-3h-containing hemolymph (mean±SE). Different letters above bars mean significant differences (*P* < 0.05, one-way ANOVA: LSD test).

The data of mortalities from different dilutions were arcsine-root transformed and tested with homogeneity of variance. The results showed that the data could be analyzed using one-way ANOVA (*P*=0.316). Differences in the mortalities of *S. litura* larvae were significant between different dilutions based on one-way ANOVA and the post-hoc analysis (*F*_7, 16_=230.75, *P*<0.0001; Figure 2). Mortalities from 10^0^ to 10^7^-fold dilutions were 98.3±1.0%, 96.5±1.0%, 98.2±1.0%, 96.5±1.0%, 69.0±3.0%, 36.7±3.0%, 10.0±3.0% and 6.0±1.0%, respectively (three repeats each dilution and thirty larvae per repeat). Larval mortalities did not differ between dilutions in 10^0^-, 10^1^-, 10^2^ and 10^3^-fold (Figure 2). However, mortalities dropped significantly from 10^3^- to 10^6^-fold dilutions (Figure 2), whereas mortalities between 10^6^- and 10^7^-fold dilutions showed no significant difference (Figure 2). Since this assay was only conducted in *S. litura* larvae, response of other noctuid species to HvAV-3h infection was further investigated.

The three noctuid larvae were all highly susceptible to 10-fold dilutions HvAV-3h-containing hemolymph infection (Table 1). No difference was detected in the larval mortalities between the three insect species (*df*=7, χ^2^=0.78, *P*=0.9976).

**Table 1:** Mortality (%) caused by HvAV-3h.

	HvAV-3h treated	Untreated control
*H. armigera*	94.4±3.7	0
*S. exigua*	88.9±3.7	0±0.12
*S. litura*	94.4±1.5	0

Notes: Values are mean (±SE) of three replicates with thirty larvae per replicate. The treated and untreated larvae were inoculated with the 10-fold dilution of the HvAV-3h-containing hemolymph and 10-fold dilutions of hemolymph from healthy larvae, respectively.

### Survival time and molting period

**Figure 3:**
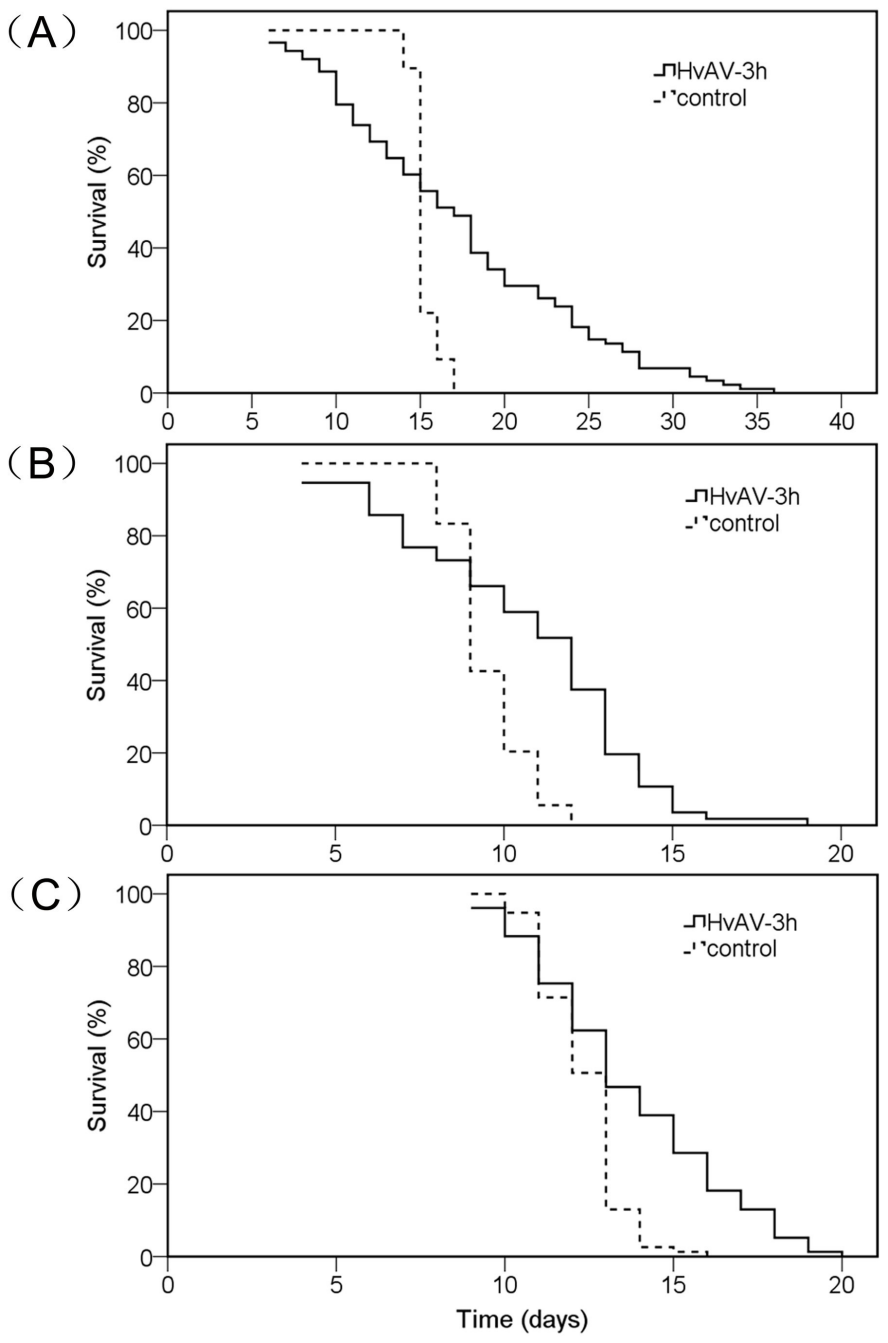
Survival curves of three insect pests larvae obtained through Kaplan-Merier with log-rank test (SPSS). *Helicoverpa armigera* (A), *Spodoptera exigua* (B) and *Spodoptera litura* (C). Control larvae were detected from inoculating time to pupating time (the end of larval stage). HvAV-3h treated larvae were detected from inoculating time to death. Treated larvae that pupated were not included in the plot.

To compare the immature stage (includes egg stage, larval stage, pre-pupal stage and pupal stage), the life spans of experimental individuals were recorded from the inoculating time point to adults’ emergence or larval death. Median survival time (ST_50_) of the infected *H. armigera*, *S.* exigua and *S. litura* larvae showed significant difference between treated and untreated groups (Table 2). Survival curve of untreated *H. armigera* larvae dropped rapidly during day 14 to day 16 post-inoculation. The curve indicated that nearly 70 percent untreated larvae finished larval stage (ending in adults’ emergence) at day 15 and less than 20 percent did within day 16 to day 17. However, the curve of treated larvae dropped progressively from day 6 to day 36 post-inoculation. The curve suggested that the mortality is roughly equally distributed from day 6 to day 36. The two curves of *H. armigera* differed significantly (Log Rank χ^2^=73.30; *df*=1; *P*<0.0001; Figure 3A).

**Table 2:** Days within larval stage of treated and untreated larvae.

	*H. armigera*		*S. exigua*		*S. litura*	
	ST_50_	Range	ST_50_	Range	ST_50_	Range
HvAV-3h	17.0±0.9	6-36	12.0±0.6	4-19	14.2±0.2	9-20
Control	15.0±0.7	14-17	9.0±0.2	8-12	16.9±0.2	10-16
Sig.	3.9820×10^-5^		7.8725×10^-6^		6.8296×10^-6^	

Notes:ST_50_ means median survival time in HvAV-3h treated larvae. Range means actual number of days infected larvae survived, i.e., a range of 6-36 means at day 6 post-inoculation, death was firstly found, at day 36 post-inoculation, death was lastly found. Sig. means significant difference between groups (Log Rank, *df*= 1, α<0.05).

The curves of other two insect species untreated control *S. eixgua* larvae dropped during 8-12 days post-inoculation, suggesting that the larvae ceased feeding and finished pupating within 8-12 days post-inoculation. The curve of treated larvae declined gradually from day 4 to day 16 post-inoculation. A straight line and a slight drop during day 16 to day 19 indicated the death of last HvAV-3h infected larvae. The two curves differed significantly (Log Rank χ^2^=19.96; *df*=1; *P*<0.0001; Figure 3B). Untreated (control) *S. litura* larvae pupated mainly between days 9 to 16 post-inoculation. The curve of treated larvae, on the other hand, dropped from day 9 to day 20, and the decrease rate (%) was similar among days. The two curves differed significantly as well (Log Rank χ^2^=20.24; *df*=1; *P*<0.0001; Figure 3 C).

We found that the longer larval stage in *H. armigera* was coherent to the longer molting period. To further test this explanation and to check whether the explanation could still be adjusted in *S. exigua* and *S. litura*, we analyzed days between 4^th^ instar (L4) and 5^th^ instar (L5) of the significantly longer-living larvae in the three species, the results showed that the days between L4 and L5 of treated *S. litura* and *S. exigua* larvae was similar to the untreated cohorts, no significant differences between treated and untreated control larvae (Table 3). On the other hand, the treated *H. armigera* larvae demonstrated increased length in instar duration (twice as many days as the untreated group in average), and differed significantly. To further understand the changes of larval growth and development among different species, we recorded data of the feeding and mass of larvae and made comparison between treated and untreated control groups.

**Table 3:** Days of the 4th instar affected by HvAV-3h.

	*H. armigera*	*S. exigua*	*S. litura*	
HvAV-3h	3.6±0.9a	1.6±0.6a	1.6±0.6a	
Control	1.5±0.5b	1.8±04a	1.8±0.4a	
*N*	54	43	49	
*P*	1.0111×10^-10^	0.3495	0.1878	

Notes: Values are mean (±SE). Different letters following the means indicate significant differences (*P* < 0.05, one-way ANOVA: LSD test)

### Larval body weight and feeding

The changes in body weight and food intake (evaporation rate 4.0% was taken into consideration) integrated the impact of HvAV-3h on larval growth and development. Untreated control larvae completed development and ceased feeding by day 7 post-inoculation in *H. armigera* and *S. exigua*, while *S. litura* in day 10 depending on species. Accordingly, the body weight and the food intake of untreated control larvae grew significantly fast with the time. On the other hand, treated larvae grew more slowly and failed to pupate. The body weight of treated larvae slightly increased within a few days after inoculation, and did not continue to go up. The food intake of treated larvae changed a little with the time. Based on the significant difference analysis, the comparison between untreated and treated group was further investigated.

**Figure 4:**
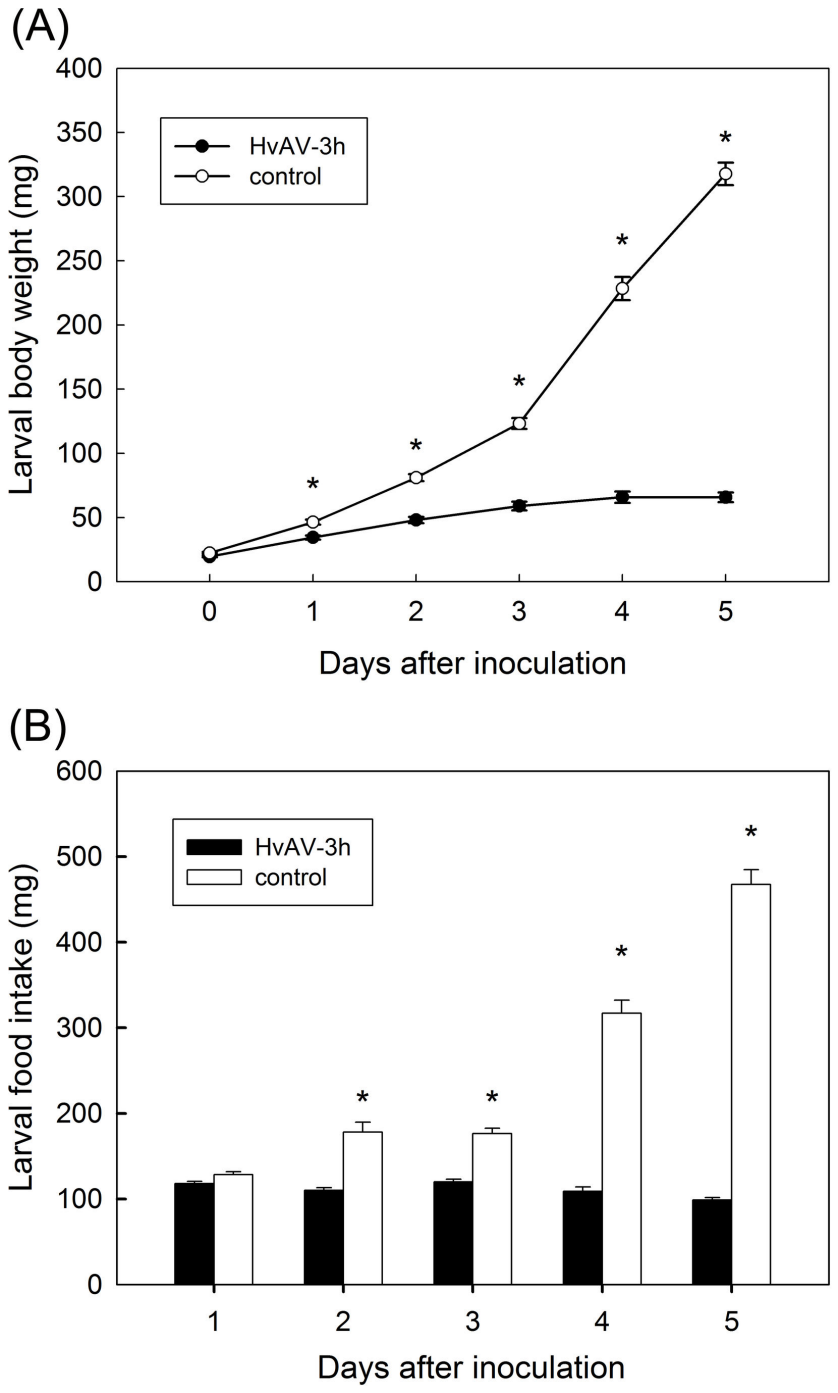
Daily body weight and food intake (mean ±SE) in *Helicoverpa armigera* larvae from L3 to pre-pupation. *Infected treatment differs significantly with untreated control (*P* < 0.05, one-way ANOVA).

**Figure 5:**
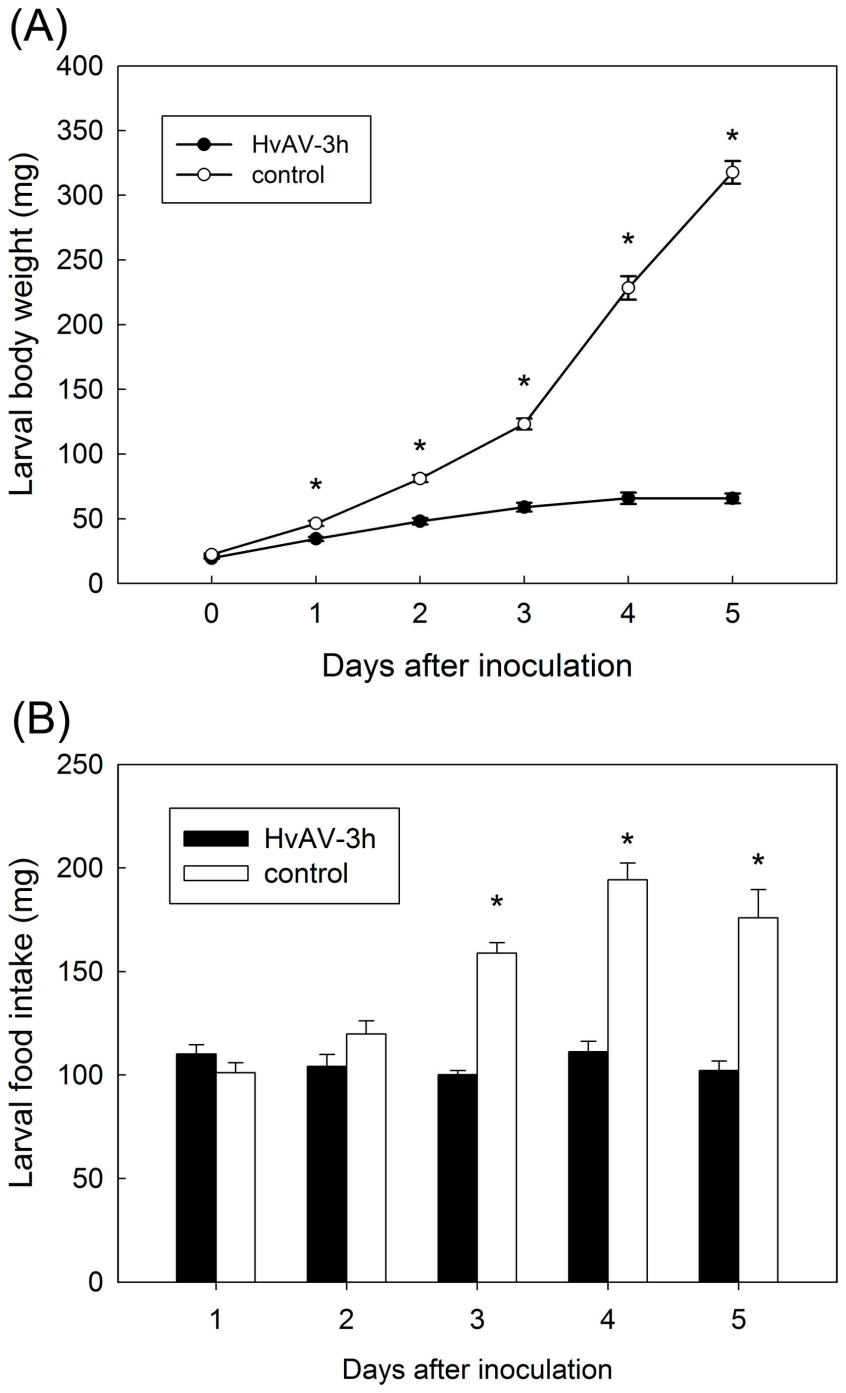
Daily body weight and food intake (mean ±SE) in *Spodoptera exigua* larvae from L3 to pre-pupation. *Infected treatment differs significantly with untreated control (*P* < 0.05, one-way ANOVA).

**Figure 6:**
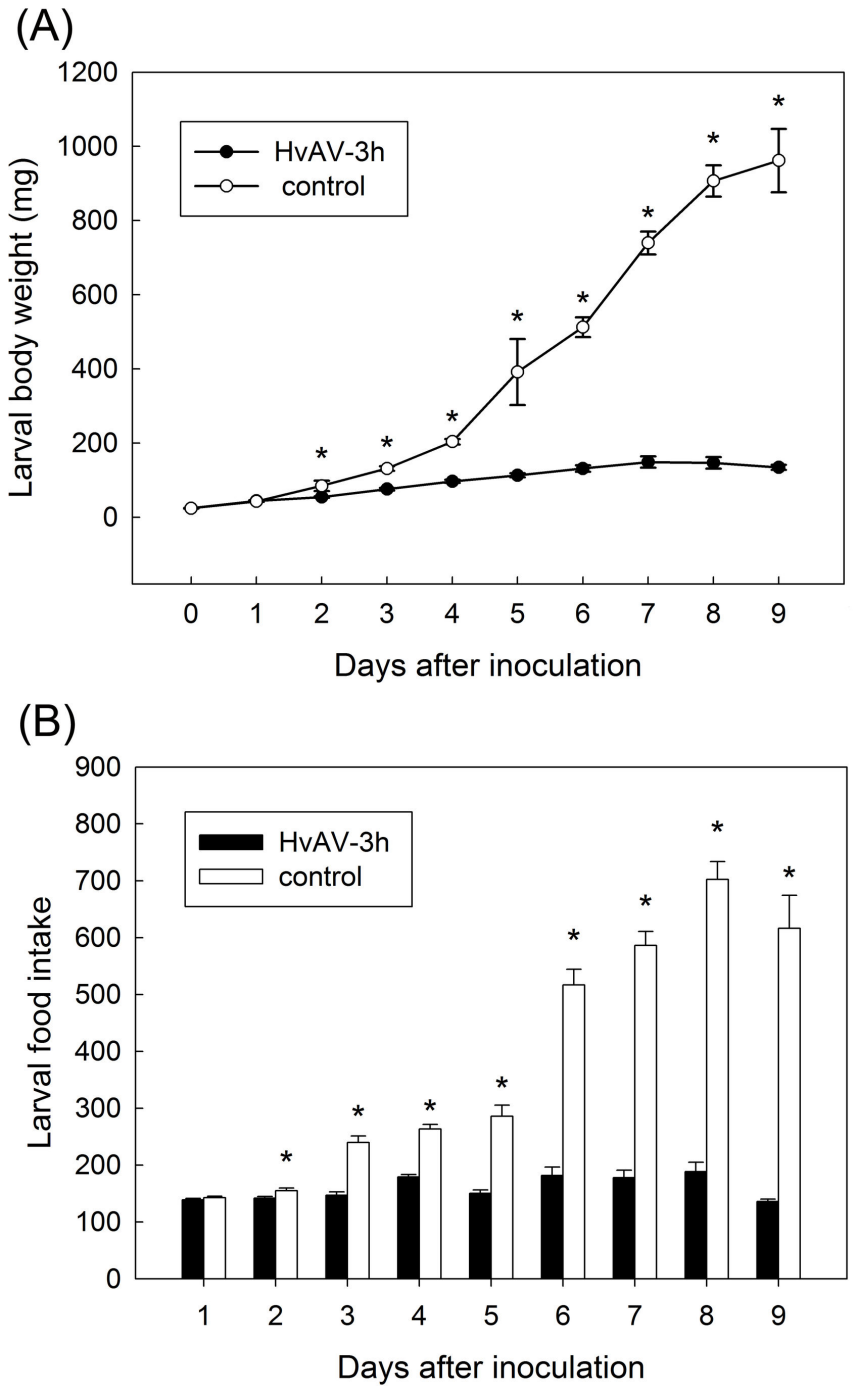
Daily body weight and food intake (mean ±SE) in *Spodoptera litura* larvae from L3 to pre-pupation. *Infected treatment differs significantly with untreated control (*P* < 0.05, one-way ANOVA).

No difference was observed in the larval body weight of *H. armigera* treated and control groups at the time of inoculation (F_1, 177_=0.97, P=0.3257; Figure 4A). From day 1 to day 5, the differences in body weight increased between the untreated larvae, that develop normally, and the treated larvae, that gained little weight. The differences between the two groups were significant from day 1 (Figure 4A) (day 1: F_1, 177_=21.85, P<0.0001; day 2: F_1, 177_=80.30, P<0.0001; day 3: F_1, 177_=139.90, P<0.0001; day 4: F_1, 177_=250.58, P<0.0001; day 5: F_1, 177_=704.53, P<0.0001). Similar trends were observed for the two other species, *S. exigua* (Figure 5A) and *S litura* (Figure 6A). Although, in *S. exigua*, no difference in body weight was detected between the treated and untreated group (*F*_1, 160_=0.05, *P*=0.0828; Figure 5A), the differences between the treated and untreated control group continued to increase as incubation time extended (day 1: *F*_1, 160_=5.71, *P*=0.0183; day 2: *F*_1, 160_=13.97, *P*<0.0001; day 3: *F*_1, 160_=70.41, *P*<0.0001; day 4: *F*_1, 160_=155.67, *P*<0.0001; day 5: *F*_1, 160_=13.423, *P*<0.0001; Figure 5A). In *S. litura*, the body weight of untreated control rose from 20 mg to 200 mg within the first four days, whereas the growth of treated group (rose from 20 mg to nearly 80 mg) was slow. Therefore, larval body weight showed a significant difference between the two treatments from day 2 to day 4 (day 2: *F*_1, 176_=4.57, *P*=0.0337; day 3: *F*_1, 176_=61.02, *P*<0.0001; day 4: *F*_1, 176_=148.63, *P*<0.0001; Figure 6A). This difference continued significantly from day 5 to day 9 (day 5: *F*_1, 176_=9.61, *P*=0.0023; day 6: *F*_1, 176_=182.14, *P*<0.0001; day 7: *F*_1, 176_=293.30, *P*<0.0001; day 8: *F*_1, 176_=351.38, *P*<0.0001; day 9: *F*_1, 176_=302.40, *P*<0.0001). No differences were detected between the treated and control larvae on the 1st-3rd days (day 0: *F*_1, 176_=0.03, *P*=0.8741; day 1: *F*_1, 176_=0.22, *P*=0.6429).

The differences in growth are correlated with differences in food consumption for the three species. The data are summarized in Figures 4B, 5B and 6B for the three species *H. armigera*, *S.* exigua and *S. litura*, respectively. In *H. armigera*, the amount of food consumed by treated larvae, on the other hand, with the exception on the 1st day (*F*_1, 177_=6.47, *P*=0.0119), was significantly lower than the untreated control larvae (day 2: *F*_1, 177_=31.07, *P*<0.0001; day 3: *F*_1, 177_=65.03, *P*<0.0001; day 4: *F*_1, 177_=163.97, *P*<0.0001; day 5: *F*_1, 177_=462.23, *P*<0.0001; Figure 4B). Untreated control larvae were gluttonous before pre-pupation (300 mg to 500 mg in average) but treated larvae showed no increment of food intake during this period of time. In *S. exigua*, the growth of food intake of untreated control group was related to body weight from day 1 to day 4 but not day 5 (control larvae digested less food in day 5 before pupation). The amounts of food consumed by treated larvae were not significantly different in day 1 and day 2 (day 1: *F*_1, 160_=1.91, *P*=0.1691; day 2: *F*_1, 160_=3.41, *P*=0.0674), but in day 3 to day 5 compared to untreated control larvae (day 3: *F*_1, 160_=120.38, *P*<0.0001; day 4: *F*_1, 160_=77.82, *P*<0.0001; day 5: *F*_1, 160_=40.18, *P*<0.0001; Figure 5B). In *S. litura*, the food intakes of both the treated and untreated groups were in accord with body weight. The food intake of *S. litura* demonstrated a significant difference compared to untreated larvae on day 2 to day 9 (day 2: *F*_1, 176_=5.54, *P*=0.0198; day 3: *F*_1, 176_=49.37, *P*<0.0001; day 4: *F*_1, 176_=87.86, *P*<0.0001; day 5: *F*_1, 176_=43.76, *P*<0.0001; day 6: *F*_1, 176_=137.29, *P*<0.0001; day 7: *F*_1, 176_=221.07, *P*<0.0001; day 8: *F*_1, 176_=223.39, *P*<0.0001; day 9: *F*_1, 176_=219.25 *P*<0.0001). However, the amount of food intake in treated larvae showed almost the same with the control larvae on day 1 (*F*_1, 176_=0.97, *P*=0.3282; Figure 6B).

## Discussion

The effects of ascovirus infection on noctuid larval physiology, including extended larval stages and difficulty in molting, have been documented in the literature [11]. However, the responses of different noctuid species to AV infection have not been systematically investigated. Accordingly, we assessed the different physiological responses to HvAV-3h infection in three noctuid species, providing information for further understanding of the possible physiological changes in larvae after AV infection.

HvAV-3h viral concentration was estimated from the total number of genomes using the end-point dilution method. The result obtained (1.1×10^11^ infectious genomes/ml) are not very different from those previously published for other ascoviruses with the titers of 8.0×10^7^ to 1.5×10^8^ vesicles/ml in ascovirus-infected *T. ni* larvae and 5.0×10^8^ vesicles/ml in *S. frugiperda* at day 5 post-inoculation [11]. The difference of viral concentration in the hemolymph was probably due to the fitness of the virus in the host. An end-point dilution approach coupled to PCR was used to determine virus concentration by the first time. But in fact, whether all virions are infectious or not was remained un-tested. It is possible some HvAV-3h form replication defective virus during cell infection, because missing of DNA polymerase in budded virus leading to defective baculovirus has been detected [29]. If this is true, our titer of HvAV-3h might be over-estimated. Also, although we determined the concentration of HvAV-3h of an inoculum, the variance of volume that entered the insect was remained un-detectable.

We showed that low HvAV-3h doses (dilutions of 10^6^-10^7^ of the stock) still caused mortality in the tested larvae. This suggests that a few viral particles would be enough to colonize a host (Figure 2). Our data support earlier reports of low dose DpAV-4a caused mortality in *Acrolepiopsis assectella* [16,30]. 

Noctuid larval mortalities caused by different concentration of virus may vary among isolates. Here, the mortalities of other reported ascovirus isolates and *S. litura* induced by HvAV-3h were compared. The larval mortality (L3 and L4) caused by Sf 82-126 (then proven to be SfAV-1a) ranged from 90.0% to 96.0% at a concentration from 10 to 10^5^ vesicles inoculated per larva without significant difference. The mortality obtained from the *T. ni* isolate (now refers to as TnAV-2a) [19], was lower, at 90.0-93.3% and varying with the vesicle content (10-10^5^ vesicles inoculated per larva). Different AVs showed no significant difference in mortalities with different concentration of AV vesicles. Mortalities of *S. litura* larvae cause by HvAV-3h showed no significant difference among undiluted virion-containing hemolymph, 10-, 10^2^-, and 10^3^- fold dilution. Our result supported the previous studies [19].

The three species, *H. armigera*, *S.* exigua and *S. litura* were with high susceptibility with HvAV-3h, and the mortalities of the three species caused by HvAV-3h did not differ significantly from each other. However, the mortalities caused by other HvAV-3 isolate varied in several noctuid larval species. To date, only HvAV-3g (isolated from Indonesia) has been evaluated for susceptibility to noctuid insects among HvAV-3 species [15]. HvAV-3g infections followed different patterns in five noctuid species, *Pseudoplusia includens*, *H. virescens*, *T. ni*, *S.* frugiperda, and also *S. exigua*. Except for *H. virescens*, the virus infected all these species. The mortality of *S. exigua* was the highest, at 100.0% in larval stage. In *P. includens*, mortalities were observed in the larval stage (28.3%) and the pre-pupal stage (46.7%). In *T. ni*, mortalities were also found at 45.4% in the larval stage, 13.6% in the pre-pupal stage, and in the pupal stage (41.0%). In *S. frugiperda*, mortalities were 86.2% in the larval stage and 13.8% in the pupal stage. The results were quite different from ours. The two *Spodoptera* species in our study were affected by HvAV-3h only in larval stage, but *S. frugiperda* larvae (belongs to *Spodoptera* species) died of HvAV-3g in pupal stage [15]. 

The ST (survival time) ranges for *S. litura*, *S.* exigua caused by HvAV-3h were smaller than those reported for other virus-infected species (8-36 d for TnAV-2a, 20-50 d for SfAV-1a in L3 larvae and 13-37 d in L4 larvae) [6]. The different reactions among species may account for different infection rates, virion production and possibly different tissue tropism by different isolates. So, the relative longevity of *H. armigera* (6-36 d) might be caused by the virus replicating mainly in the tracheal epithelium tissue and epidermal cells, but not replicating in vital tissues [6]. Because the previous study did not address the virulence of HvAV-3a in other species, we cannot say in which tissue the virus replicates.

Two insect hormones, juvenile hormone and ecdysone regulate larval development, the former maintains the larval status and the latter promotes ecdysis [31-33]. Larval molting is another index reflecting changes in hormone balance induced by HvAV-3h or even reflecting larval metabolism during growing stages [19,34,35]. HvAV-3h is able to alter larval molting period, this is another important feature of AV infection in larvae [14,19]. In *H. armigera*, in contrast to the untreated control larvae, most *H. armigera* larvae showed an extended larval instar period, over 3 days in average (Table 3). Infection of HvAV-3h may be similar to that caused by polydnaviruses, which reduce ecdysone synthesis, thus prolonging larval development [15,35]. Based on our experiments, HvAV-3h infection in *H. armigera* larvae may either produce a viral protein(s) that sequesters ecdysone to extend the instars or the infection reduces the production of ecdysone, thus leading to long period of instar development time. The validity of this explanation, however, remains to be tested. 

The most conspicuous physiological change in noctuid larvae infected with HvAV-3h was the body weight and food intake over time comparing with control larvae. Body weight is a fundamental indication involving larval growth and development [36-38]. Specifically, the body weight in infected larvae rose very slowly and showed the significant difference with the untreated group from the first/second day post-inoculation. The significant difference of food intake between groups, on the other hand, was observed from the second (third) day post-inoculation. Our results supported previous studies [14,19]. 

Over all, HvAV-3h has pronounced physiological impact on *H. armigera*, *S.* exigua and *S. litura*, and the three insect pest species cause significant crop loss in agriculture. Thereafter, our results suggest that HvAV-3h could play a role in the control of pest populations based on its function during ecological regulation, in other words, an important factor that help to maintain the stabilization and equilibrium of the ecosystem. Firstly, AVs, including HvAV-3h, can be transmitted in the field from ascovirus-infected larvae to healthy larvae by parasitic wasps during oviposition [10,17,19,39-41]. Secondly, dose-response results indicated that successful infection could be obtained even when larvae are inoculated with low doses of virus; so, transmission via parasitoid wasps would not be limited to high concentration of virus [16]. These two reasons make transmission of HvAV-3h via parasitoid wasps an attractive hypothesis to be tested in the future. Furthermore, we found that HvAV-3h has a more rapid killing speed compared to other reported AVs [11,15,19] with the ability to immediately reduce larval feeding, weight and thus inhibit metamorphosis, namely, larvae infected with HvAV-3h would not have gluttony, as a result, the crop yield loss would be less with infected larvae. However, the technical barriers such as the viable infection method and the viral durable capability should be figured out before HvAV-3h could be put into use in the agricultural condition.
